# Impacto dos Bloqueios Intraventriculares na Dinâmica do Ciclo Cardíaco: Uma Análise ECO e Vetorcardiográfica

**DOI:** 10.36660/abc.20240253

**Published:** 2024-11-27

**Authors:** Carlos Eduardo Duarte, Katia Regina Silva, Luciene Dias de Jesus, Henry Abensur, Roberto Costa

**Affiliations:** 1 Hospital das Clínicas Faculdade de Medicina Universidade de São Paulo São Paulo SP Brasil Instituto do Coração do Hospital das Clínicas da Faculdade de Medicina da Universidade de São Paulo, São Paulo, SP – Brasil; 2 Beneficência Portuguesa de São Paulo São Paulo SP Brasil Beneficência Portuguesa de São Paulo, São Paulo, SP – Brasil

**Keywords:** Bloqueio de Ramo, Eletrocardiografia, Vetorcardiografia, Terapia de Ressincronização Cardíaca

## Abstract

**Fundamento:**

O bloqueio do ramo esquerdo (BRE) provoca retardos que alteram a mecânica do ciclo ventricular. O efeito de outros bloqueios intraventriculares (BIV) permanece pouco explorado.

**Objetivos:**

Estudar as fases do ciclo cardíaco (CC) e a sincronia ventricular em diferentes padrões de ativação ventricular.

**Métodos:**

Estudo transversal com 328 indivíduos consecutivos sem cardiopatia estrutural, eletrocardiograma normal ou BIV, realizado entre agosto/2020 e janeiro/2022. Ecocardiograma e Vetorcardiograma foram realizados simultaneamente para analisar a eletromecânica do CC. Foi uitlizada a Análise de Variância de um fator (ANOVA) com teste de comparação múltipla de Bonferroni, com nível de significância de 5%.

**Resultados:**

A idade dos participantes foi de 64,8±15,3 anos, com 57,9% do sexo masculino e fração de ejeção de 67,0±6,8%. O eletrocardiograma foi normal em 32,3%, 18,6% apresentavam bloqueio do ramo direito (BRD), 17,7% apresentavam bloqueio da divisão anterossuperior (BDAS), 15,6% apresentavam BRD+BDAS e 15,9% apresentavam BRE. No ecocardiograma, verificou-se aumento da pré-ejeção do ventrículo esquerdo de 18,7% (p<0,001) e 56,8% (p<0,001) no BRD+BDAS e BRE, respectivamente. Houve contração miocárdica pós-sistólica em todos os tipos de BIV e dissincronia ventricular no BRE. Pelo Vetorcardiograma, observou-se ativação inicial da onda R aumentada em 17,4% no BDAS (p<0,001), 43,5% no BRD+BDAS (p<0,001) e 47,4% no BRE (p<0,001) e final atrasada em 69,4% no BRE (p<0,001), 73,6% no BRD+BDAS (p<0,001) e 95,3% no BRD (p<0,001).

**Conclusões:**

Todos BIV modificaram o CC; entretanto, apenas o BRE e o BRD+BDAS alteraram significativamente o ciclo ventricular esquerdo, evidenciando a maior complexidade desses distúrbios.

## Graphical abstract

**Figura Central:**
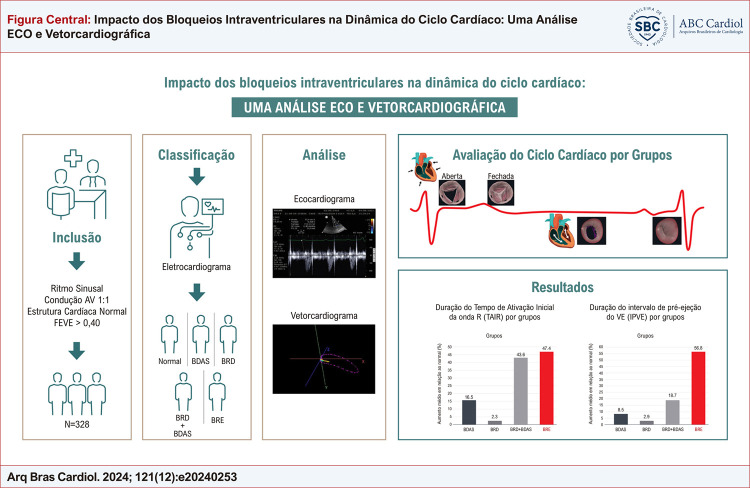
: Impacto dos Bloqueios Intraventriculares na Dinâmica do Ciclo Cardíaco: Uma Análise ECO e Vetorcardiográfica

## Introdução

Distúrbios da condução elétrica intraventricular estão associados a retardo da ativação miocárdica, alteração da sincronia ventricular normal e perda da eficiência mecânica do coração.^1^ Esses fenômenos podem variar em grau e em localização, assim como acometer global ou regionalmente um único ou ambos os ventrículos, dependendo do tipo de distúrbio elétrico.^2^ O estudo dos efeitos dos bloqueios da condução intraventricular sobre a função ventricular ganhou maior interesse a partir do desenvolvimento da terapia de ressincronização cardíaca (TRC) e da observação de que seus efeitos clínicos e funcionais variavam em função do tipo de bloqueio.^3^

O eletrocardiograma de superfície tem sido o método utilizado para classificar os diversos tipos de bloqueios da condução intraventricular, enquanto o ecocardiograma, nas suas diversas modalidades, tem permitido avaliar os efeitos funcionais desses distúrbios elétricos.^4^ Recentemente, a transformação matemática dos sinais do eletrocardiograma de superfície tornou possível a obtenção do vetorcardiograma de forma simplificada.^5^ Este método aumenta a acurácia do ECG para determinar os atrasos da ativação elétrica e da repolarização cardíaca por permitir a análise vetorial simultânea em três planos, além da decomposição das alças elétricas das ondas P e T e dos complexos QRS em suas fases inicial e final, através da identificação dos seus pontos de amplitude máxima.^6^

A avaliação dos efeitos funcionais do bloqueio completo do ramo esquerdo no ciclo cardíaco (CC) tem demonstrado o aumento da duração da sístole e a diminuição da diástole, dentre outros efeitos deletérios à dinâmica do ventrículo esquerdo (VE) causados por este distúrbio elétrico.^7^ Contudo, no melhor do nosso conhecimento, esses fenômenos ainda não foram estudados nos demais tipos de bloqueio intraventricular. A justificativa para o presente estudo baseia-se na necessidade de se identificar quais tipos de bloqueios intraventriculares (BIV) de padrão não-BRE estão associados a alterações significativas do ciclo ventricular esquerdo e, que, portanto, podem se beneficiar da TRC.

O objetivo do presente estudo foi avaliar, pelo vetorcardiograma e pelo ecocardiograma, indivíduos com diferentes tipos de condução intraventricular, para compreender as alterações elétricas e suas correspondências mecânicas nas diferentes fases do CC.

## Métodos

### Desenho do estudo e aspectos éticos

Estudo transversal realizado em indivíduos consecutivos referenciados para realização de ecocardiograma bidimensional de rotina em um centro terciário de cardiologia no período de agosto de 2020 a janeiro de 2022. O estudo foi aprovado pelo Comitê de Ética em Pesquisa da Instituição. Todos os participantes assinaram o termo de consentimento livre e esclarecido (TCLE).

### População estudada

Foram incluídos indivíduos adultos, com coração morfologicamente normal, ritmo sinusal regular, condução atrioventricular 1:1 e condução intraventricular normal ou com padrões eletrocardiográficos de bloqueio da divisão anterossuperior (BDAS), bloqueio do ramo direito (BRD), bloqueio do ramo direito com bloqueio divisional anterossuperior (BRD+BDAS) ou bloqueio do ramo esquerdo (BRE). Não foram incluídos indivíduos com histórico de doença cardíaca estrutural, especialmente defeito cardíaco congênito, doença de Chagas ou infarto do miocárdio; de procedimentos cirúrgicos ou percutâneos cardiovasculares prévios; ablação de arritmias cardíacas ou uso de dispositivo cardíaco eletrônico implantável; uso de medicamentos que retardam a despolarização cardíaca ou alteram o intervalo QT como antiarrítmicos ou antidepressivos; áreas eletricamente inativas no eletrocardiograma ou fração de ejeção menor do que 0,40; alteração da contratilidade segmentar; ou áreas de cicatrizes ventriculares ao ecocardiograma.

A amostra, definida por conveniência, foi composta pelos indivíduos que realizam o ecocardiograma bidimensional de rotina de maneira consecutiva no período do estudo e que atenderam aos critérios de elegibilidade.

Foram excluídos do estudo os casos que apresentaram inviabilidade técnica para a aquisição de medidas ecocardiográficas específicas ou para geração das alças vetorcardiográficas devido à baixa qualidade do sinal eletrocardiográfico, assim como os que apresentaram alterações eletrocardiográficas ou ecocardiográficas indicativas de modificações na contratilidade segmentar, áreas eletricamente inativas ou sinais de disfunção ventricular com fração de ejeção do ventrículo esquerdo (FEVE) < 0,40.

### Avaliação dos dados clínicos basais

Após a inclusão no estudo, foi realizado o levantamento do histórico cardiovascular e do estado funcional, mediante consulta do prontuário e entrevista com os pacientes.

### Estudo ecocardiográfico

As medidas temporais do CC foram determinadas a partir do início do complexo QRS e visaram o cálculo do intervalo de pré-ejeção, do período ejetivo, do tempo de relaxamento isovolumétrico e do tempo de enchimento do VE, assim como do intervalo de pré-ejeção e do período ejetivo do ventrículo direito (VD). A sincronia interventricular foi determinada pela diferença entre os intervalos pré-ejetivos das câmaras ventriculares direitas e esquerdas e a sincronia intraventricular, pela diferença temporal entre os nadires das paredes lateral e septal obtidos durante a avaliação da contratilidade ventricular esquerda pelo modo M. A presença de contração muscular após o fechamento valvar aórtico (contração diastólica) foi mensurada para as paredes septal e lateral.

### Estudo eletrocardiográfico

A categorização morfológica dos padrões de ativação intraventricular, foi feita por um único analisador em conformidade com os seguintes critérios: (1) Grupo NORMAL, caracterizado por complexo QRS < 120 ms com ângulo máximo do complexo QRS no plano frontal (SâQRS) entre -30° e 90°; (2) Grupo BDAS incluindo complexo QRS < 120 ms com desvio do eixo no plano frontal para a esquerda com SâQRS < -45° com padrão rS em D2 e D3, sendo D3 de 15 mm e D3>D2; (3) Grupo BRD compreendendo complexo QRS > 120 ms e presença de onda R trifásica em V1 ou V2 (rsr’, rsR’, rSR’); (4) Grupo BRD+BDAS caracterizado pela associação dos padrões de BRD e BDAS acima descritos e (5) Grupo BRE incluindo complexo QRS > 120 ms na presença onda R bifásica em V1 ou V2 (QS ou rS).^8^

As medidas da frequência cardíaca (ciclo RR), da duração do complexo QRS e dos intervalos PR e QT foram realizadas de forma automática pelo software do equipamento utilizado para o registro do ECG e conferidas manualmente para a certificação de sua precisão. A correção do intervalo QT pela frequência cardíaca foi realizada pelo software seguindo a fórmula de Bazzett.^9^ O SâQRS foi anotado após a definição correta dos intervalos acima mencionados. A unidade de tempo utilizada foi o milissegundo (ms), a unidade de amplitude foi o microvolt (µV) e a unidade angular foi o grau (°).

### Vetorcardiograma

**Figura 1:**
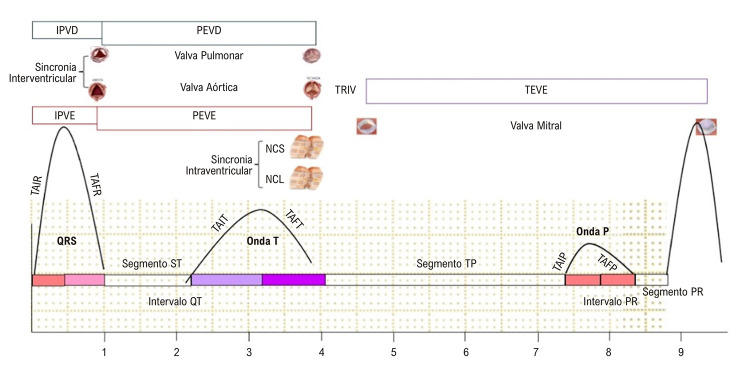
– Representação esquemática da avaliação vetorecocardiográfica do ciclo cardíaco. IPVD: intervalo de pré-ejeção do ventrículo direito; IPVE: intervalo de pré ejeção do ventrículo esquerdo; NCL: nadir da contração da parede lateral; NCS: nadir da contração da parede Septal; PEVD: período ejetivo do ventrículo direito; PEVE: período ejetivo do ventrículo esquerdo; TAFP: tempo de ativação final da onda P; TAFR: tempo de ativação final da onda R; TAFT: tempo de ativação final da onda T; TAIP: tempo de ativação inicial da onda P; TAIR: tempo de ativação inicial da onda R; TAIT: tempo de ativação inicial da onda T; TEVE: tempo de enchimento do ventrículo esquerdo; TRIV: tempo de relaxamento isovolumétrico.

Os sinais vetorcardiográficos, construídos pela transformação matemática dos sinais eletrocardiográficos seguindo a regra de Kors,^10^permitiram a determinação dos tempos de ativação inicial e final da onda R, dos tempos de ativação inicial e final da onda T, dos tempos de ativação inicial e final da onda P nas alças tridimensionais (3-D), construídas pela soma vetorial dos planos frontal, horizontal e sagital e dos Segmentos isoelétricos ST, TP e PR (Figura 1).

### Coleta eletrônica e gerenciamento dos dados

A coleta de dados foi realizada em formulários eletrônicos desenvolvidos no software REDCap (*Research Electronic Data Capture*).^11^ Ao longo do estudo, foram utilizadas funcionalidades específicas do REDCap para o monitoramento da qualidade dos dados.

### Variáveis estudadas e análise estatística

Para a análise dos resultados foram considerados os dados clínicos basais, os dados do eletrocardiograma, do ecocardiograma e do vetorcardiograma. Para as variáveis contínuas esta análise foi feita pela apresentação do cálculo de médias e desvios-padrão. Para as variáveis categóricas calculou-se frequências absolutas e relativas. O teste de Kolmogorov-Smirnov (KS) foi utilizado para a testagem da normalidade dos dados.

Para a comparação dos grupos foi realizada a Análise de Variância de um fator com teste de comparação múltipla de Bonferroni. Para se testar a associação entre as proporções foi utilizado o teste qui-quadrado ou o teste exato de Fisher, conforme a natureza dos dados. O nível de significância utilizado para os testes foi de 5%.

## Resultados

### Características basais dos participantes

Durante o período do estudo, 394 indivíduos foram avaliados. Destes, 57 não foram incluídos por apresentar histórico de infarto do miocárdio ou por estar em uso de medicamentos que alteram o segmento ST ou o intervalo QT. Após a inclusão no estudo, nove participantes foram excluídos por apresentar, ao ecocardiograma, alteração segmentar da contratilidade cardíaca ou fração de ejeção do VE <0,40 ou por problemas técnicos para a obtenção do vetorcardiograma. Desta forma, a amostra do estudo foi composta por 328 indivíduos, distribuídos conforme os critérios eletrocardiográficos estabelecidos para a condução intraventricular: NORMAL em 106 (32,3%), BDAS em 58 (17,7%), BRD em 61 (18,6%), BRD+BDAS em 51 (15,6%) e BRE em 52 (15,9%) participantes. As características basais da amostra estão apresentadas na Tabela 1.

**Tabela 1:** – Características basais dos participantes da pesquisa segundo os grupos estudados

Variáveis estudadas	Todos (n = 328)	NORMAL (n = 106)	BDAS (n = 58)	BRD (n = 61)	BRD+BDAS (n = 51)	BRE (n = 52)	p
**Idade (anos), média ± DP**	64,9 ± 15,3	55,2 ± 16,2	69,8 ± 11,1	68,2 ± 14,6	71,8 ± 10,6	67,3 ± 12,5	< 0,001
**Sexo, n (%)**							
Feminino	138 (42,0)	55 (51,9)	20 (34,5)	22 (36,1)	15 (29,4)	26 (50,0)	0,025
Masculino	190 (57,9)	51 (48,1)	38 (65,5)	39 (63,9)	36 (70,6)	26 (50,0)	
**Classe Funcional (NYHA), n (%)**							
I	323 (98,5)	106 (100)	58 (100)	60 (98,4)	51 (100)	48 (92,3)	0,003
II	5 (1,5)	0 (0,0)	0 (0,0)	1 (1,6)	0 (0,0)	4 (7,7)	
**Comorbidades, n (%)**							
Hipertensão arterial	186 (56,7)	44 (41,5)	39 (67,2)	38 (62,3)	32 (62,8)	33 (63,5)	0,005
Dislipidemia	128 (39,0)	30 (28,3)	24 (41,4)	24 (39,3)	19 (37,3)	31 (59,6)	0,006
Diabetes	89 (27,1)	21 (19,8)	18 (31,0)	19 (31,2)	13 (25,5)	18 (34,6)	0,252
Hipotireoidismo	43 (13,1)	13 (12,3)	3 (5,2)	6 (9,8)	10 (19,6)	11 (21,2)	0,069
**Medicamentos, n (%)**							
Uso de qualquer medicamento	256 (78,1)	62 (58,5)	50 (86,2)	52 (85,3)	46 (90,2)	46 (88,5)	<0,001
IECA/BRA	154 (60,2)	30 (48,4)	35 (70,0)	32 (61,5)	27 (58,7)	30 (65,2)	0,186
Betabloqueador	64 (25,0)	13 (21,0)	10 (20,0)	10 (19,2)	11 (23,9)	20 (43,5)	0,032
Furosemida	11 (4,3)	1 (1,6)	1 (2,0)	0 (0,0)	4 (8,7)	5 (10,9)	0,019
Espironolactona	13 (5,1)	1 (1,6)	2 (4,0)	0 (0,0)	0 (0,0)	10 (21,7)	<0,001
**Variáveis Ecocardiográficas, média ± DP**							
Átrio Esquerdo (mm)	35,5 ± 4,7	34,0 ± 3,7	34,9 ± 4,2	35,5 ± 5,4	37,8 ± 4,6	36,9 ± 5,3	< 0,001
Ventrículo Esquerdo (mm)	47,9 ± 4,9	46,7 ± 4,8	48,2 ± 4,6	47,6 ± 4,5	49,3 ± 5,1	49,3 ± 5,5	0,004
Ventrículo direito (mm)	25,8 ± 3,0	25,2 ± 2,7	25,3 ± 2,8	26,4 ± 3,2	27,0 ± 3,2	25,9 ± 2,9	0,002
Fração de Ejeção (%)	67,0 ± 6,8	67,4 ± 6,0	67,9 ± 5,4	69,5 ± 4,7	67,1 ± 5,5	61,8 ± 9,9	<0,001

BRA: bloqueadores do receptor de angiotensina II; DP: desvio-padrão; IECA: inibidores da conversão da enzima da angiotensina; NYHA: New York Heart Association.

### Avaliação ecocardiográfica do ciclo cardíaco

Os momentos de abertura e de fechamento das valvas pulmonar, aórtica e mitral estão apresentados no Material Suplementar (Tabela S1).

Houve alterações significativas do intervalo de pré-ejeção e do tempo de relaxamento isovolumétrico do VE associadas aos bloqueios da condução intraventricular (Tabela 2). Nota-se que os padrões de BRD e de BDAS, isoladamente, não estiveram associados a aumento significativo da pré-ejeção ventricular esquerda em relação ao grupo NORMAL. Por outro lado, no subgrupo BRD+BDAS houve aumento de 18,7% no período de pré-ejeção e no BRE, aumento de 56,8%. Vale ressaltar que o período ejetivo do VE não foi comprometido em nenhum dos tipos de BIV estudados. O tempo de relaxamento isovolumétrico apresentou aumento significativo associado apenas ao padrão BRD+BDAS. Não foram observadas alterações significativas no enchimento VE associadas aos BIV.

**Tabela 2:** – Caracterização das fases da sístole e diástole segundo os grupos estudados

Variáveis estudadas	Todos (n = 328)	NORMAL (n = 106)	BDAS (n = 58)	BRD (n = 61)	BRD+BDAS (n = 51)	BRE (n = 52)	p
**Sístole – Ventrículo Esquerdo**							
Intervalo de pré-ejeção do VE	100,8 ± 25,9	88,4 ± 16,3	95,9 ± 20,1	91,1 ± 19,5	105,0 ± 22,6	138,7 ± 20,1	<0,001
Tempo de ejeção do VE	300,3 ± 33,6	298,8 ± 32,8	306,4 ± 35,3	295,7 ± 34,2	298,8 ± 34,7	303,5 ± 31,6	0,427
**Diástole – Ventrículo Esquerdo**							
Tempo de relaxamento isovolumétrico	87,5 ± 34,9	79,3 ± 29,2	86,3 ± 37,6	85,8 ± 30,9	100,2 ± 42,2	95,3 ± 35,1	0,004
Tempo de enchimento do VE	473,4 ± 131,8	472,3 ± 136,7	475,2 ± 140,51	473,9 ± 123,2	509,4 ± 135,1	437,4 ± 112,0	0,102
**Sístole – Ventrículo Direito**							
Intervalo de pré-ejeção do VD	106,5 ± 27,3	91,0 ± 17,6	99,5 ± 25,7	125,9 ± 18,7	132,4 ± 24,2	97,88 ± 25,59	<0,001
Tempo de ejeção do VD	306,4 ± 38,7	308,7 ± 34,5	312,7 ± 39,6	299,4 ± 37,1	307,1 ±40,2	302,00 ± 45,29	0,329

BDAS: bloqueio divisional anterossuperior; BRD: bloqueio de ramo direito; BRE: bloqueio de ramo esquerdo; VD: ventrículo direito; VE: ventrículo esquerdo.

O intervalo de pré-ejeção do VD também apresentou alterações associadas aos BIVs (Tabela 2). Nota-se que, em comparação à condução NORMAL, os padrões BRD e BRD+BDAS estiveram associados a aumento significativo dos intervalos de pré-ejeção do VD, de 38,5% para o BRD e de 45,5% para o BRD+BDAS, não tendo sido observadas alterações significativas nos padrões BDAS e BRE. Quanto ao período de ejeção do VD, não houve modificação significativa associada a qualquer dos distúrbios da condução.

**Figura 2:**
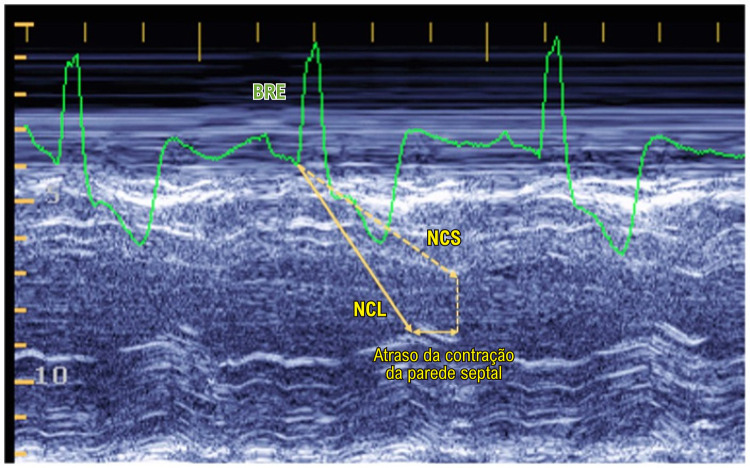
– Atraso do nadir da contração septal em indivíduo portador de Bloqueio de Ramo esquerdo. BRE: bloqueio de ramo esquerdo; NCL: nadir da contração da parede lateral; NCS: nadir da contração da parede septal.

Dissincronia interventricular foi verificada em todos os tipos de BIV com complexo QRS largo. Enquanto nos indivíduos com padrão de condução NORMAL ou com BDAS a abertura da valva pulmonar ocorreu logo após a da valva aórtica, a ocorrência de BRD ou de BRD+BDAS implicou em ampliar essa diferença. Por outro lado, o BRE esteve associado a retardo significativo do início da ejeção do VE. Dissincronia intraventricular foi observada apenas no padrão BRE, no qual a parede septal atingiu seu nadir notavelmente após o da parede lateral (Figura 2). Em todos os demais padrões de BIV a sincronia das paredes septal e lateral do VE foi pouco alterada (Tabela 3).

**Tabela 3:** – Avaliação da sincronia ventricular e da contração diastólica segundo os grupos estudados

Variáveis estudadas	Todos (n = 328)	NORMAL (n = 106)	BDAS (n = 58)	BRD (n = 61)	BRD+BDAS (n = 51)	BRE (n = 52)	p
Sincronia interventricular (ms)	5,7 ± 32,2	2,5 ± 18,8	3,5 ± 24,9	34,9 ± 19,8	27,4 ± 19,9	-40,8 ± 25,2	< 0,001
**Tempo transcorrido após o fechamento da valva aórtica**							
Nadir de contração da parede Septal (ms)	28,7 ± 70,1	-1,7 ± 56,4	25,2 ± 75,5	34,5 ± 53,2	34,3 ± 61,8	82,4 ± 80,8	< 0,001
Nadir de contração da parede Lateral (ms)	13,3 ± 47,2	8,1 ± 35,9	7,4 ± 47,6	23,6 ± 45,0	22,0 ± 61,2	9,8 ± 52,5	< 0,001
Sincronia entre as paredes lateral e septal do VE (ms)	-15,4 ± 76,4	9,9 ± 58,5	-17,7 ± 84,1	-10,9 ± 65,0	-12,2 ± 82,0	-72,6 ± 78,9	< 0,001

BDAS: bloqueio divisional anterossuperior; BRD: bloqueio de ramo direito; BRE: bloqueio de ramo esquerdo; VD: ventrículo direito; VE: ventrículo esquerdo.

Contração ventricular durante o período de relaxamento isovolumétrico, contudo, foi observada em todos os padrões de condução intraventricular estudados. A única condição em que o nadir da contração ocorreu durante a fase de ejeção ventricular foi no padrão de condução normal da parede septal. Em todas as demais condições, o nadir da contração ocorreu após o fechamento da valva aórtica, inclusive no padrão de condução normal na parede lateral do VE, sendo importante notar que, em todos os padrões de BIV estudados, a contração mais tardia foi sempre a da parede septal (Tabela 3).

### Avaliação vetorcardiográfica do ciclo cardíaco

Os valores médios das fases de ativação inicial e final, assim como as amplitudes cúbicas, das ondas P, T e complexo QRS, e a duração dos segmentos PR, ST e TP, para cada um dos subgrupos estudados, estão apresentados na Tabela 4.

**Tabela 4:** – Intervalos de avaliação vetorcardiográfica segundo os grupos estudados

Variáveis estudadas	Todos (n = 328)	NORMAL (n = 106)	BDAS (n = 58)	BRD (n = 61)	BRD+BDAS (n = 51)	BRE (n = 52)	p
**Tempo de Ativação da onda P (ms)**							
Inicial	56,4 ± 12,2	57,4 ± 10,0	55,7 ± 13,3	54,4 ± 12,3	57,7 ± 12,6	56,2 ± 14,3	0,520
Final	61,0 ± 15,8	56,5 ± 10,9	62,6 ± 19,0	63,5 ± 16,3	65,8 ± 16,6	61,1 ± 17,1	< 0,001
**Tempo de Ativação da onda R (ms)**							
Inicial	54,6 ± 14,7	46,4 ± 4,8	54,0 ± 10,3	47,4 ± 15,1	66,6 ± 18,7	68,4 ± 8,6	< 0,001
Final	74,9 ± 25,3	53,0 ± 7,9	56,2 ± 11,9	103,5 ± 19,3	92,0 ± 19,4	89,8 ± 10,6	< 0,001
**Tempo de Ativação da onda T (ms)**							
Inicial	97,1 ± 14,2	95,7 ± 13,5	99,6 ± 17,6	95,3 ± 11,8	97,1 ± 15,9	99,6 ± 11,9	0,239
Final	95,8 ± 18,7	86,2 ± 12,0	93,6 ± 13,9	95,2 ± 12,3	101,8 ± 16,7	112,8 ± 27,4	< 0,001
**Amplitude cúbica (µV)**							
Onda P	136,7 ± 37,9	138,3 ± 33,0	132,4 ± 40,0	141,0 ± 45,2	140,8 ± 40,1	128,9 ± 32,5	0,342
Complexo QRS	1010,4 ± 407,7	1081,7 ± 355,7	866,7 ± 292,5	818,1 ± 274,9	779,8 ± 306,6	1477,1 ± 418,4	< 0,001
Onda T	365,6 ± 138,7	352,2 ± 124,8	333,6 ± 115,2	385,0 ± 135,0	336,4 ± 129,1	434,3 ± 176,1	< 0,001
**Segmentos isoelétricos**							
Segmento PR (ms)	49,0 ± 23,7	42,9 ± 18,5	51,2 ± 17,8	50,0 ± 31,3	51,7 ± 27,1	55,1 ± 22,9	0,018
Segmento ST (ms)	97,9 ± 34,6	119,0 ± 29,7	107,2 ± 27,3	81,4 ± 31,7	86,1 ± 33,7	75,5 ± 27,4	< 0,001
Segmento TP (ms)	308,8 ± 132,8	330,0 ± 125,1	329,0 ± 137,7	290,0 ± 121,9	310,2 ± 155,8	263,8 ± 120,3	0,023

BDAS: bloqueio divisional anterossuperior; BRD: bloqueio de ramo direito; BRE: bloqueio de ramo esquerdo; VD: ventrículo direito; VE: ventrículo esquerdo.

O tempo de ativação inicial do complexo QRS não se mostrou alterado em relação ao padrão normal somente pelo BRD. Nos demais padrões, houve retardo da ativação ventricular de 17,4% no BDAS, de 43,5% no BRD+BDAS e de 47,4% no BRE. O tempo de ativação final do complexo QRS não foi alterado significativamente apenas no BDAS. Apresentou-se aumentado em relação ao Normal em todos os outros tipos de BIV estudados, com incremento médio de 69,4% no BRE, de 73,6% no BRD+BDAS e de 95,3% no BRD (Tabela 4).

A repolarização ventricular também foi alterada pelos bloqueios da condução intraventricular com modificações observadas na duração do segmento ST, na duração da onda T e em sua simetria. O segmento ST não se mostrou significativamente alterado apenas no padrão BDAS. Nos outros padrões de BIV houve encurtamento do segmento ST que, em média, foi de 36,0% no BRE, de 32,0% no BRD e de 28,0% no BRD+BDAS. A duração da fase inicial da onda T não se mostrou significativamente alterada em nenhum dos padrões de BIV estudados. Por outro lado, a porção final da onda T mostrou-se com duração aumentada em todos os padrões de BIV, com incremento médio de 8,6% no BDAS (p=0,465), de 10,4% no BRD (p<0,001), de 18,1% no BRD+BDAS (p<0,001) e de 30,9% no BRE (p<0,001). Estas alterações descritas causaram, portanto, modificações da simetria das ondas T (Tabela 4).

Os segmentos TP e PR dos grupos de pacientes com distúrbio da condução IV não apresentaram modificações significativas quando comparados ao grupo NORMAL à exceção do grupo BRE, em que a duração do intervalo TP foi 20% menor (p=0,031) e a duração do segmento PR foi 28% maior do que o NORMAL (p=0,022).

A ativação atrial mostrou-se semelhante em todos os padrões de condução intraventricular, tendo sido notada diferença em relação ao grupo NORMAL apenas no tempo de ativação final da onda P no subgrupo BRD+BDAS em que se notou incremento médio de 17,0% (p=0,004).

## Discussão

As alterações da contratilidade cardíaca causadas por distúrbios da condução intraventricular têm sido estudadas desde os anos 1960.^12,13^ No entanto, foi somente em 1989 que Grines et al.,^7^ descreveram as modificações ventriculares globais resultantes da ativação elétrica anormal do BRE, evidenciadas principalmente pela movimentação anormal do septo interventricular, redução da fração de ejeção regional do VE e redução da diástole. Essas descobertas embasaram a TRC, que visa a redução de efeitos deletérios à função ventricular esquerda induzidos por bloqueios da condução intraventricular ou pela estimulação elétrica artificial crônica do VD. A alta taxa de não respondedores à TRC dos pacientes com padrão não-BRE; entretanto, destaca a necessidade de uma maior compreensão das modificações ocorridas no CC e na sincronia das paredes ventriculares desses indivíduos, a fim de evitar que pacientes com baixa probabilidade de resposta sejam submetidos à TRC ou que indivíduos com maior probabilidade de boa resposta não recebam esse tratamento.^13^

O presente estudo visou avaliar o CC em quatro padrões de bloqueio da condução intraventricular para identificar as alterações elétricas e mecânicas que ocorrem em comparação ao padrão de condução normal. As principais alterações da temporização identificadas incluíram aumento do intervalo de pré-ejeção dos ventrículos, assincronia interventricular, assincronia intraventricular esquerda e presença de contração ventricular esquerda durante a fase de relaxamento isovolumétrico.

A importância do aumento do intervalo de pré-ejeção do VE causado pelos BIVs já foi demonstrada em estudos prévios, inclusive, como preditor da resposta à TRC.^14^ Neste estudo, observou-se que o aumento do período de pré-ejeção do VE foi a principal alteração detectada no CC, estando principalmente associado ao BRE e, em menor intensidade, ao BRD+BDAS, não estando associado ao BDAS ou ao BRD. A despeito das modificações detectadas na pré-ejeção, não foram identificadas alterações significativas no tempo de ejeção em nenhum dos padrões eletrocardiográficos estudados. Além disso, não foram observadas alterações significativas do tempo de diástole ventricular esquerda, em contraste com os achados anteriormente reportados.^7^

A falta de sincronia entre as contrações dos ventrículos direito e esquerdo, embora seja frequentemente utilizada como critério para indicação da TRC,^15^ nos indivíduos com padrão BRD ou BRD+BDAS sinalizou, apenas, o retardo da abertura da valva pulmonar, não tendo sido notadas consequências para o ciclo do VE. Por outro lado, a avaliação da sincronia intraventricular esquerda pela medida do tempo do nadir das contrações das paredes septal e lateral do VE demonstrou que, embora apenas os indivíduos com BRE apresentassem dissincronia significativa entre essas paredes, em todos os padrões de BIV estudados, o nadir das contrações de ambas as paredes analisadas ocorria após o fechamento da valva aórtica, portanto, durante a fase de diástole do VE.

As modificações na ativação elétrica ventricular detectadas pelo vetorcardiograma mostraram que, a despeito do aumento global do tempo de duração dos complexos QRS no BRD, no BRD+BDAS e no BRE, apenas os padrões BRD+BDAS e BRE apresentaram um aumento significativo no tempo de ativação inicial. No padrão BRD, o aumento na duração do complexo QRS ocorreu, exclusivamente, pelo aumento do tempo de ativação final do complexo QRS, embora os padrões BRE e BRD+BDAS também tenham apresentado aumento significativo da fase de ativação final. As alterações na repolarização ventricular associadas aos BIVs foram caracterizadas pelo encurtamento do segmento ST nos padrões BRD, BRD+BDAS e BRE e por modificações na duração e simetria das ondas T.

As alterações elétricas e mecânicas identificadas neste estudo, ao mesmo tempo que confirmam os graves impactos do BRE para o ciclo e para a sincronia ventricular esquerda, demonstram que o BDAS e o BRD isolado estão associados a modificações menos relevantes nesta câmara cardíaca. Por outro lado, a associação BRD+BDAS merece ser avaliada com um número amostral mais representativo, face à grande diversidade de resultados detectados tanto pelo vetorcardiograma quanto pela ecocardiografia.

A análise da demografia e da apresentação clínica da amostra estudada apresenta diferenças nítidas entre indivíduos com padrão eletrocardiográfico normal e aqueles com BIV. Observa-se uma idade média mais elevada dos subgrupos de indivíduos com BIVs e a maior prevalência do sexo masculino nos indivíduos com os padrões BDAS, BRD e BRD+BDAS. Entretanto, à semelhança do que ocorre nos indivíduos com padrão de condução intraventricular normal, aqueles com padrão de BRE apresentaram equilíbrio na proporção entre os sexos. Quanto à prescrição de medicamentos, foi possível verificar o uso significativamente maior de fármacos de ação cardiovascular nos indivíduos com padrão BRE.

Os dados clínicos, vetorcardiográficos e morfológicos deste estudo destacam a maior gravidade das repercussões do BRE, sugerindo que os pacientes com esse padrão eletrocardiográfico, mesmo na ausência de sinais ou de sintomas de insuficiência cardíaca ou de disfunção ventricular esquerda grave, devem receber atenção especial durante o seguimento clínico, tanto para a prescrição de medicamentos que evitem o agravamento da função ventricular esquerda quanto para a indicação da TRC quando o tratamento farmacológico não resultar em melhora clínica e da função ventricular esquerda. Além disso, nossos resultados mostram a importância da avaliação do CC em pacientes candidatos à TRC.

### Limitações do estudo

As alterações do CC avaliadas no presente estudo referem-se a indivíduos sem evidência de cardiopatia estrutural ou de alterações do ritmo cardíaco manifestadas por arritmia atrial ou bloqueio avançado da condução atrioventricular. Esses achados precisam ser validados em outras condições clínicas, como infarto do miocárdio prévio, disfunção ventricular esquerda, disfunções valvares graves, ou em outras condições que não foram contempladas nos critérios de inclusão deste estudo.

## Conclusões

A análise dos quatro padrões de distúrbios da condução intraventricular mostrou associações significativas com alterações no CC e na sincronia ventricular, variando em grau de localização conforme o tipo de bloqueio. A principal alteração observada no CC foi o aumento do período de pré-ejeção do VE associada tanto ao BRE quanto ao BRD+BDAS. Apenas o padrão BRE apresentou associação significativa com dissincronia intraventricular esquerda.
