# Supplementary material for: Impacto dos Bloqueios Intraventriculares na Dinâmica do Ciclo Cardíaco: Uma Análise ECO e Vetorcardiográfica
Source: Arq Bras Cardiol. 2024 Nov 27;121(12):e20240253. [Article in Portuguese] doi: 10.36660/abc.20240253 (PMC11634305; doi:10.36660/abc.20240253)
Supplement: Translation

# Translation: Impact of Intraventricular Blocks on Cardiac Cycle Dynamics: An Echocardiographic and Vectorcardiographic Analysis

**DOI:** 10.36660/abc.20240253i

**Authors:** Carlos Eduardo Duarte, Katia Regina Silva, Luciene Dias de Jesus, Henry Abensur, Roberto Costa

**Affiliations:** 1 Brazil Instituto do Coração do Hospital das Clínicas da Faculdade de Medicina da Universidade de São Paulo, São Paulo, SP – Brazil; 2 Brazil Beneficência Portuguesa de São Paulo, São Paulo, SP – Brazil

**Keywords:** Bundle-Branch Block, Electrocardiography, Vectorcardiography, Cardiac Resynchronization Therapy

## Abstract

**Background:**

Left bundle branch block (LBBB) causes delays that alter the mechanics of the ventricular cycle. The effect of other intraventricular blocks (IVB) remains little explored.

**Objectives:**

To study the phases of the cardiac cycle (CC) and ventricular synchrony in different ventricular activation patterns.

**Methods:**

Cross-sectional study with 328 consecutive individuals without structural heart disease, normal electrocardiogram or IVB, conducted in the period between August/2020 and January/2022. Echocardiogram and Vectorcardiogram were performed simultaneously to analyze the electromechanics of the CC. A one-way Analysis of Variance (ANOVA) with Bonferroni’s multiple comparison test was used, with a significance level of 5%.

**Results:**

The age of the participants was 64.8±15.3 years, with 57.9% male and an ejection fraction of 67.0±6.8%. The electrocardiogram was normal for 32.3%, 18.6% had right bundle branch block (RBBB), 17.7% had left anterior fascicular block (LAFB), 15.6% had RBBB+LAFB, and 15.9% had LBBB. The echocardiogram showed an increased left ventricular pre-ejection by 18.7% (p<0.001) and 56.8% (p<0.001) in RBBB+LAFB and LBBB, respectively. There was a post-systolic myocardial contraction in all types of IVB and ventricular dyssynchrony in LBBB. Using the vectorcardiogram, initial activation of the R wave was increased by 17.4% in LAFB (p<0.001), 43.5% in RBBB+LAFB (p<0.001) and 47.4% in LBBB (p<0.001) and delayed final activation by 69.4% in LBBB (p<0.001), 73.6% in RBBB+LAFB (p<0.001) and 95.3% in RBBB (p<0.001).

**Conclusion:**

All IVBs modified the CC; however, only LBBB and RBBB+LAFB significantly changed the left ventricular cycle, thereby evidencing the greater complexity of these disorders.

## Introduction

Disturbances in intraventricular electrical conduction are linked to delayed myocardial activation, disruption of normal ventricular synchrony, and a reduction in the heart’s mechanical efficiency.^1^ These effects can vary in degree and location, affecting either globally or regionally, one or both ventricles, depending on the type of electrical disturbance.^2^ Interest in the study of intraventricular conduction blocks and their impact on ventricular function has grown significantly since the introduction of resynchronization therapy (CRT) and the realization that its clinical and functional outcomes differ based on the type of block.^3^

The surface ECG has traditionally been used to classify the various types of intraventricular conduction blocks, while echocardiography, in its various modalities, has made it possible to evaluate the functional impact of these electrical disturbances.^4^ Recently, the mathematical transformation of surface ECG signals has enabled a simplified method for obtaining the vectorcardiogram.^5^ This approach enhances the accuracy of the ECG in identifying delays in electrical activation and cardiac repolarization by allowing simultaneous vector analysis in three planes, as well as decomposition of the electrical loops for the P and T waves and the QRS complexes in both their initial and final phases, identifying points of maximum amplitude.^6^

Assessments of the functional effects of complete LBBB during the cardiac cycle (CC) have demonstrated an extension of systole and a reduction of diastole, along with other harmful effects on the left ventricle (LV) dynamics caused by this electrical disturbance.^7^ However, to the best of our knowledge, these phenomena have yet to be examined in other types of intraventricular blocks. The rationale for this study stems from the need to identify which types of non-LBBB pattern IVB are linked to significant alterations in the LV cycle and may, therefore, benefit from CRT.

This study aimed to evaluate, using vectorcardiography and echocardiography, individuals with different types of intraventricular conduction in order to understand the electrical alterations and their mechanical correspondence during the various phases of the CC.

## Methods

### Study design and ethical aspects

This was a cross-sectional study conducted on consecutive individuals referred for routine two-dimensional echocardiography at a tertiary cardiology center between August 2020 and January 2022. The Research Ethics Committee of the Institution approved the study. All participants signed the informed consent form (ICF).

### Population studied

Adult individuals with a morphologically normal heart, regular sinus rhythm, 1:1 atrioventricular conduction and normal intraventricular conduction or with electrocardiographic patterns of Left anterior fascicular block (LAFB), right bundle branch block (RBBB), right bundle branch block with Left anterior fascicular block (RBBB+LAFB) or left bundle branch block (LBBB) were included. Exclusion criteria included individuals with a history of structural heart disease, such as congenital heart defects, Chagas disease, or myocardial infarction; those with prior cardiovascular surgical or percutaneous procedures; prior ablation of cardiac arrhythmias or use of implantable electronic cardiac devices; use of medications that affect cardiac depolarization or alter the QT interval, such as antiarrhythmics or antidepressants; presence of electrically inactive areas on the ECG; ejection fraction lower than 0.40; segmental contractility alterations; or evidence of ventricular scarring on echocardiography.

The sample was defined by convenience, composed of individuals who underwent routine two-dimensional echocardiography consecutively during the study period and who met the eligibility criteria.

Cases where it was technically impossible to obtain specific echocardiographic measurements or to generate vectorcardiographic loops due to poor ECG signal quality were excluded, as well as those with ECG or echocardiographic signs of segmental contractility abnormalities, electrically inactive areas, or evidence of ventricular dysfunction with left ventricular ejection fraction (LVEF) < 0.40.

### Assessment of baseline clinical data

After inclusion in the study, the cardiovascular history and functional status were assessed by consulting the medical records and interviewing the patients.

### Echocardiographic study

The temporal measurements of the CC were determined from the onset of the QRS complex and aimed to calculate the pre-ejection interval, ejection period, isovolumetric relaxation time, and LV filling time, as well as the pre-ejection interval and ejection period of the right ventricle (RV). Interventricular synchrony was assessed by the difference between the pre-ejection intervals of the right and left ventricles. In contrast, intraventricular synchrony was determined by the temporal difference between the nadirs of the lateral and septal walls during M mode evaluation of left ventricular contractility. Muscle contraction following aortic valve closure (diastolic contraction) was measured in the septal and lateral walls.

### Electrocardiographic study

A single analyzer performed the morphological categorization of intraventricular activation patterns in accordance with the following criteria: (1) NORMAL group, characterized by QRS complex < 120 ms with a maximum angle of the QRS complex in the frontal plane (SâQRS) between -30° and 90°; (2) LAFB group including QRS complex < 120 ms with deviation of the axis in the frontal plane to the left with SâQRS < -45° with rS pattern in D2 and D3, with D3 of 15 mm and D3>D2; (3) RBBB group comprising QRS complex > 120 ms and presence of triphasic R wave in V1 or V2 (rsr’, rsR’, rSR’); (4) RBBB+LAFB group characterized by the association of the RBBB and LAFB patterns described above and (5) LBBB group including QRS complex > 120 ms in the presence of biphasic R wave in V1 or V2 (QS or rS).^8^

The measurements of heart rate (RR cycle), QRS complex duration and PR and QT intervals were performed automatically by the software of the equipment used to record the ECG and checked manually to certify their accuracy. The correction of the QT interval by heart rate was performed via the software following the Bazzett formula.^9^ The SâQRS was recorded after the correct definition of the aforementioned intervals. The time unit used was millisecond (ms), the amplitude unit was microvolt (µV), and the angular unit was degree (°).

### Vectorcardiogram

The vectorcardiographic signals, built by the mathematical transformation of the electrocardiographic signals following the Kors rule,^10^ allowed the determination of the initial and final activation times of the R wave, initial and final activation times of the T wave, initial and final activation times of the P wave in the three-dimensional (3-D) loops, as built by the vector sum of the frontal, horizontal and sagittal planes and the isoelectric segments ST, TP, and PR (Figure 1).

**Figure 1:** – Schematic representation of the vectorechocardiographic evaluation of the cardiac cycle. RVPEI: right ventricular pre-ejection interval; LVPEI: left ventricular pre-ejection interval; NLC: nadir of lateral wall contraction; NSC: nadir of septal wall contraction; RVEP: right ventricular ejection period; LVEP: left ventricular ejection period; FATP: final activation time of the P Wave; FATR: final activation time of the R Wave; FATT: final activation time of the T Wave; IATP: initial activation time of the P Wave; IATR: initial activation time of the R Wave; IATT: initial activation time of the T Wave; LVFT: left ventricular filling time; IVRT: isovolumetric relaxation time.

### Electronic Collection and Data Management

Data collection was performed on electronic forms developed in the REDCap (Research Electronic Data Capture) software.^11^ Throughout the study, specific REDCap functionalities were used to monitor data quality.

### Variables and Statistical Analysis

Baseline clinical data, electrocardiogram, echocardiogram and vectorcardiogram data were considered for the analysis of the results. For continuous variables, this analysis was performed by calculating means and standard deviations. For categorical variables, absolute and relative frequencies were calculated. The Kolmogorov-Smirnov (KS) test was used to test data normality.

One-way analysis of variance with Bonferroni’s multiple comparison test was performed to compare the groups. To test the association between proportions, the chi-square test or Fisher’s exact test was used, depending on the nature of the data. The significance level used for the tests was 5%.

## Results

### Participants’ baseline characteristics

During the study period, 394 individuals were evaluated. Of these, 57 were not included due to a history of myocardial infarction or due to taking medications that alter the ST segment or QT interval. After inclusion in the study, nine participants were excluded due to echocardiogram findings showing segmental alterations in cardiac contractility or LV ejection fraction <0.40 or due to technical problems in obtaining the vectorcardiogram. Thus, the study sample consisted of 328 individuals, distributed according to the electrocardiographic criteria established for intraventricular conduction: NORMAL in 106 (32.3%), LAFB in 58 (17.7%), RBBB in 61 (18.6%), RBBB+LAFB in 51 (15.6%) and LBBB in 52 (15.9%) participants. The baseline characteristics of the sample are presented in Table 1.

**Table 1:** – Baseline characteristics of research participants according to the groups studied

Variables	All (n = 328)	NORMAL (n = 106)	LAFB (n = 58)	RBBB (n = 61)	RBBB+LAFB (n = 51)	LBBB (n = 52)	p
**Age (years), mean ± SD**	64.9 ± 15.3	55.2 ± 16.2	69.8 ± 11.1	68.2 ± 14.6	71.8 ± 10.6	67.3 ± 12.5	< 0.001
**Sex, n (%)**							
Female	138 (42.0)	55 (51.9)	20 (34.5)	22 (36.1)	15 (29.4)	26 (50.0)	0.025
Male	190 (57.9)	51 (48.1)	38 (65.5)	39 (63.9)	36 (70.6)	26 (50.0)	
**Functional Class (NYHA), n (%)**							
I	323 (98.5)	106 (100)	58 (100)	60 (98.4)	51 (100)	48 (92.3)	0.003
II	5 (1.5)	0 (0.0)	0 (0.0)	1 (1.6)	0 (0.0)	4 (7.7)	
**Comorbidities, n (%)**							
Arterial hypertension	186 (56.7)	44 (41.5)	39 (67.2)	38 (62.3)	32 (62.8)	33 (63.5)	0.005
Dyslipidemia	128 (39.0)	30 (28.3)	24 (41.4)	24 (39.3)	19 (37.3)	31 (59.6)	0.006
Diabetes	89 (27.1)	21 (19.8)	18 (31.0)	19 (31.2)	13 (25.5)	18 (34.6)	0.252
Hypothyroidism	43 (13.1)	13 (12.3)	3 (5.2)	6 (9.8)	10 (19.6)	11 (21.2)	0.069
**Medication, n (%)**							
Use of any medication	256 (78.1)	62 (58.5)	50 (86.2)	52 (85.3)	46 (90.2)	46 (88.5)	<0.001
ACEI/ARB	154 (60.2)	30 (48.4)	35 (70.0)	32 (61.5)	27 (58.7)	30 (65.2)	0.186
Beta-blocker	64 (25.0)	13 (21.0)	10 (20.0)	10 (19.2)	11 (23.9)	20 (43.5)	0.032
Furosemide	11 (4.3)	1 (1.6)	1 (2.0)	0 (0.0)	4 (8.7)	5 (10.9)	0.019
Spironolactone	13 (5.1)	1 (1.6)	2 (4.0)	0 (0.0)	0 (0.0)	10 (21.7)	<0.001
**Echocardiographic variables, mean ± SD**							
Left Atrium (mm)	35.5 ± 4.7	34.0 ± 3.7	34.9 ± 4.2	35.5 ± 5.4	37.8 ± 4.6	36.9 ± 5.3	< 0.001
Left Ventricle (mm)	47.9 ± 4.9	46.7 ± 4.8	48.2 ± 4.6	47.6 ± 4.5	49.3 ± 5.1	49.3 ± 5.5	0.004
Right Ventricle (mm)	25.8 ± 3.0	25.2 ± 2.7	25.3 ± 2.8	26.4 ± 3.2	27.0 ± 3.2	25.9 ± 2.9	0.002
Ejection Fraction (%)	67.0 ± 6.8	67.4 ± 6.0	67.9 ± 5.4	69.5 ± 4.7	67.1 ± 5.5	61.8 ± 9.9	<0.001

ARB: angiotensin II receptor blockers; SD: standard deviation; ACEI: angiotensin-converting enzyme inhibitors; NYHA: New York Heart Association.

### Echocardiographic assessment of the cardiac cycle

The opening and closing times of the pulmonary, aortic, and mitral valves are provided in the Supplementary Material (Table S1).

Significant changes were found in the pre-ejection interval and LV isovolumetric relaxation time, associated with intraventricular conduction blocks (Table 2). It is noteworthy that RBBB and LAFB patterns, in isolation, did not show a significant increase in LV pre-ejection compared to the NORMAL group. However, in the RBBB+LAFB subgroup, there was an 18.7% increase in the pre-ejection period, and in LBBB, an increase of 56.8%. Importantly, the LV ejection period was not affected in any of the IVB types studied. The isovolumetric relaxation time showed a significant increase only in the RBBB+LAFB pattern. No significant changes in LV filling were observed in relation to intraventricular blocks.

**Table 2:** – Characterization of the phases of systole and diastole according to the groups studied

Variables	All (n = 328)	NORMAL (n = 106)	LAFB (n = 58)	RBBB (n = 61)	RBBB+LAFB (n = 51)	LBBB (n = 52)	p
**Systole – Left Ventricle**							
LV pre-ejection interval	100.8 ± 25.9	88.4 ± 16.3	95.9 ± 20.1	91.1 ± 19.5	105.0 ± 22.6	138.7 ± 20.1	<0.001
LV ejection time	300.3 ± 33.6	298.8 ± 32.8	306.4 ± 35.3	295.7 ± 34.2	298.8 ± 34.7	303.5 ± 31.6	0.427
**Diastole – Left Ventricle**							
Isovolumetric relaxation time	87.5 ± 34.9	79.3 ± 29.2	86.3 ± 37.6	85.8 ± 30.9	100.2 ± 42.2	95.3 ± 35.1	0.004
LV filling time	473.4 ± 131.8	472.3 ± 136.7	475.2 ± 140.51	473.9 ± 123.2	509.4 ± 135.1	437.4 ± 112.0	0.102
**Systole – Right Ventricle**							
RV pre-ejection interval	106.5 ± 27.3	91.0 ± 17.6	99.5 ± 25.7	125.9 ± 18.7	132.4 ± 24.2	97.88 ± 25.59	<0.001
RV ejection time	306.4 ± 38.7	308.7 ± 34.5	312.7 ± 39.6	299.4 ± 37.1	307.1 ±40.2	302.00 ± 45.29	0.329

LAFB: left anterior fascicular block; RBBB: right bundle branch block; LBBB: left bundle branch block; RV: right ventricle; LV: left ventricle.

The RV pre-ejection interval also showed changes related to IVBs (Table 2). Compared to NORMAL conduction, RBBB, and RBBB+LAFB patterns showed a significant increase in RV pre-ejection intervals, with increases of 38.5% for RBBB and 45.5% for RBBB+LAFB, with no significant changes in LAFB or LBBB patterns. Regarding the RV ejection period, no significant change was observed in any conduction disorder.

Interventricular dyssynchrony was identified in all IVB types with wide QRS complexes. In individuals with NORMAL conduction or LAFB, the pulmonary valve opened shortly after the aortic valve. However, in cases of RBBB or RBBB+LAFB, this delay increased. On the other hand, LBBB was associated with a significant delay in the onset of LV ejection. Intraventricular dyssynchrony was observed only in the LBBB pattern, in which the septal wall reached its nadir, notably after that of the lateral wall (Figure 2). In all other IVB patterns, the synchrony of the LV septal and lateral walls was little altered (Table 3).

**Figure 2:** – Delayed septal contraction nadir in an individual with left bundle branch block. LBBB: left bundle branch block; NLC: nadir of lateral wall contraction; NSC: nadir of septal wall contraction.

**Table 3:** – Assessment of ventricular synchrony and diastolic contraction according to the groups studied

Variables	All (n = 328)	NORMAL (n = 106)	LAFB (n = 58)	RBBB (n = 61)	RBBB+LAFB (n = 51)	LBBB (n = 52)	p
Interventricular synchrony (ms)	5.7 ± 32.2	2.5 ± 18.8	3.5 ± 24.9	34.9 ± 19.8	27.4 ± 19.9	-40.8 ± 25.2	< 0.001
**Time elapsed after aortic valve closure**							
Nadir of septal wall contraction (ms)	28.7 ± 70.1	-1.7 ± 56.4	25.2 ± 75.5	34.5 ± 53.2	34.3 ± 61.8	82.4 ± 80.8	< 0.001
Nadir of lateral wall contraction (ms)	13.3 ± 47.2	8.1 ± 35.9	7.4 ± 47.6	23.6 ± 45.0	22.0 ± 61.2	9.8 ± 52.5	< 0.001
Synchronization between the lateral and septal walls of the LV (ms)	-15.4 ± 76.4	9.9 ± 58.5	-17.7 ± 84.1	-10.9 ± 65.0	-12.2 ± 82.0	-72.6 ± 78.9	< 0.001

LAFB: left anterior fascicular block; RBBB: right bundle branch block; LBBB: left bundle branch block; RV: right ventricle; LV: left ventricle.

Ventricular contraction during the period of isovolumetric relaxation, however, was observed in all intraventricular conduction patterns studied. The only condition in which the nadir of contraction occurred during the ventricular ejection phase was the normal conduction pattern of the septal wall. In all other conditions, the nadir of contraction occurred after aortic valve closure, including in the normal conduction pattern of the LV lateral wall. Notably, in all IVB patterns studied, the latest contraction always occurred in the septal wall (Table 3).

### Vectorcardiographic assessment of the cardiac cycle

The mean values of the initial and final activation phases, as well as the cubic amplitudes of the P wave, T wave and QRS complex, and the duration of the PR, ST and TP segments, for each of the subgroups studied, are shown in Table 4.

**Table 4:** – Vectorcardiographic evaluation intervals according to the groups studied

Variables	All (n = 328)	NORMAL (n = 106)	LAFB (n = 58)	RBBB (n = 61)	RBBB+LAFB (n = 51)	LBBB (n = 52)	p
**P wave activation time (ms)**							
Initial	56.4 ± 12.2	57.4 ± 10.0	55.7 ± 13.3	54.4 ± 12.3	57.7 ± 12.6	56.2 ± 14.3	0.520
Final	61.0 ± 15.8	56.5 ± 10.9	62.6 ± 19.0	63.5 ± 16.3	65.8 ± 16.6	61.1 ± 17.1	< 0.001
**R wave activation time (ms)**							
Initial	54.6 ± 14.7	46.4 ± 4.8	54.0 ± 10.3	47.4 ± 15.1	66.6 ± 18.7	68.4 ± 8.6	< 0.001
Final	74.9 ± 25.3	53.0 ± 7.9	56.2 ± 11.9	103.5 ± 19.3	92.0 ± 19.4	89.8 ± 10.6	< 0.001
**T wave activation time (ms)**							
Initial	97.1 ± 14.2	95.7 ± 13.5	99.6 ± 17.6	95.3 ± 11.8	97.1 ± 15.9	99.6 ± 11.9	0.239
Final	95.8 ± 18.7	86.2 ± 12.0	93.6 ± 13.9	95.2 ± 12.3	101.8 ± 16.7	112.8 ± 27.4	< 0.001
**Cubic amplitude (µV)**							
P wave	136.7 ± 37.9	138.3 ± 33.0	132.4 ± 40.0	141.0 ± 45.2	140.8 ± 40.1	128.9 ± 32.5	0.342
QRS complex	1010.4 ± 407.7	1081.7 ± 355.7	866.7 ± 292.5	818.1 ± 274.9	779.8 ± 306.6	1477.1 ± 418.4	< 0.001
T Wave	365.6 ± 138.7	352.2 ± 124.8	333.6 ± 115.2	385.0 ± 135.0	336.4 ± 129.1	434.3 ± 176.1	< 0.001
**Isoelectric segments**							
PR segment (ms)	49.0 ± 23.7	42.9 ± 18.5	51.2 ± 17.8	50.0 ± 31.3	51.7 ± 27.1	55.1 ± 22.9	0.018
ST segment (ms)	97.9 ± 34.6	119.0 ± 29.7	107.2 ± 27.3	81.4 ± 31.7	86.1 ± 33.7	75.5 ± 27.4	< 0.001
TP segment (ms)	308.8 ± 132.8	330.0 ± 125.1	329.0 ± 137.7	290.0 ± 121.9	310.2 ± 155.8	263.8 ± 120.3	0.023

LAFB: left anterior fascicular block; RBBB: right bundle branch block; LBBB: left bundle branch block; RV: right ventricle; LV: left ventricle.

The initial activation time of the QRS complex was not changed in relation to the normal pattern by RBBB alone. In the other patterns, there was a delay in ventricular activation of 17.4% in LAFB, 43.5% in RBBB+LAFB and 47.4% in LBBB. The final activation time of the QRS complex was not significantly changed in LAFB only. It was increased in relation to Normal in all other types of IVB studied, with an average increase of 69.4% in LBBB, 73.6% in RBBB+LAFB and 95.3% in RBBB (Table 4).

Intraventricular conduction blocks also changed ventricular repolarization with changes observed in the ST segment duration, the T wave duration, and its symmetry. The ST segment was not significantly changed in the LAFB pattern only. In the other IVB patterns, there was an average shortening of the ST of 36.0% for LBBB, 32.0% for RBBB and 28.0% for RBBB+LAFB. The duration of the initial phase of the T wave was not significantly changed in any of the IVB patterns studied. In contrast, the final portion of the T wave showed prolonged duration across all IVB patterns, with an average increase of 8.6% in LAFB (p=0.465), 10.4% in RBBB (p<0.001), 18.1% in RBBB+LAFB (p<0.001), and 30.9% in LBBB (p<0.001). These alterations caused changes in T wave symmetry (Table 4).

The TP and PR segments of the groups with IV conduction disorders showed no significant differences compared to the NORMAL group, except in the LBBB group, where the TP interval was 20% shorter (p=0.031), and the PR segment duration was 28% longer than in the NORMAL group (p=0.022).

Atrial activation was similar in all intraventricular conduction patterns, with a difference in relation to the NORMAL group only being noted in the final activation time of the P wave for the RBBB+LAFB subgroup, in which a mean increase of 17.0% was noted (p=0.004).

## Discussion

Research on changes in cardiac contractility caused by intraventricular conduction disorders dates back to the 1960s.^12,13^ However, it was not until 1989 that Grines et al.^7^ described the global ventricular alterations resulting from abnormal electrical activation in LBBB, particularly abnormal interventricular septal motion, reduced regional LV ejection fraction, and shortened diastole. These findings supported CRT, aimed at reducing the adverse effects on LV function caused by intraventricular conduction blocks or chronic artificial RV electrical stimulation. The high rate of non-responders to CRT in patients with non-LBBB patterns; however, highlights the need for a greater understanding of changes that occur in the CC and the synchrony of the ventricular walls of these individuals, in order to prevent patients with a low probability of response from undergoing CRT or individuals with a higher probability of good response from not receiving this treatment.^13^

This study aimed to evaluate the CC in four types of intraventricular conduction block to identify the electrical and mechanical changes that occur compared to the normal conduction pattern. The main timing changes identified were an increase in the pre-ejection interval of the ventricles, interventricular dyssynchrony, left intraventricular dyssynchrony, and LV contraction during the isovolumetric relaxation phase.

The significance of the increased LV pre-ejection interval due to IVBs has been demonstrated in previous studies, including its use as a predictor of CRT response.^14^ In this study, the increase in the LV pre-ejection period was the main CC alteration observed, primarily associated with LBBB and, to a lesser extent, with RBBB+LAFB, with no association seen in LAFB or RBBB. Despite the changes detected in pre-ejection, no significant changes in ejection time were observed in any of the electrocardiographic patterns studied. Furthermore, no significant changes in left ventricular diastole time were observed, in contrast to previously reported findings.^7^

The lack of synchrony between right and left ventricular contractions, although often used as a criterion for indicating CRT,^15^ in individuals with RBBB or RBBB+LAFB patterns only reflected the delay in pulmonary valve opening without affecting the LV cycle. In contrast, the evaluation of left intraventricular synchrony by measuring the nadir timing of the septal and lateral wall contractions of the LV revealed that, although significant dyssynchrony between these walls was only observed in individuals with LBBB, the nadir of contractions in both walls occurred after the closure of the aortic valve in all IVB patterns studied, which places these contractions during the LV diastole phase.

Ventricular electrical activation changes detected by vectorcardiography showed that despite the overall increase in QRS complex duration in RBBB, RBBB+LAFB, and LBBB patterns, only RBBB+LAFB and LBBB patterns presented a significant increase in initial activation time. In the RBBB pattern, the prolongation of the QRS complex was solely due to the extension of its final activation time. At the same time, LBBB and RBBB+LAFB patterns also exhibited a significant increase in the final activation phase. The ventricular repolarization changes associated with IVBs were characterized by a shortened ST-segment in RBBB, RBBB+LAFB, and RBBB patterns, along with changes in T wave duration and symmetry.

The electrical and mechanical changes identified in this study confirm the severe impacts of LBBB on the CC and LV synchrony. At the same time, LAFB and isolated RBBB were associated with less significant alterations in this cardiac chamber. On the other hand, the RBBB+LAFB combination warrants further investigation with a larger sample size due to the wide range of results detected by both vectorcardiography and echocardiography.

The analysis of the demographics and clinical presentation of the sample studied shows clear differences between individuals with a normal electrocardiographic pattern and those with IVB. There was a higher mean age in the subgroups of individuals with IVBs and a higher prevalence of males in individuals with the LAFB, RBBB, and RBBB+LAFB patterns. However, similar to individuals with a normal intraventricular conduction pattern, those with an LBBB pattern showed a balanced sex distribution. Regarding medication use, a significantly greater use of cardiovascular drugs was noted in individuals with LBBB patterns.

The clinical, vectorcardiographic, and morphological data from this study highlight the greater severity of LBBB’s effects, suggesting that patients with this electrocardiographic pattern, even without signs or symptoms of heart failure or severe LV dysfunction, should receive special attention during clinical follow-up, both for prescribing medications to prevent worsening LV function and for considering CRT when pharmacological treatment does not result in clinical or LV functional improvement. Moreover, our results show the importance of evaluating the CC in patients who are candidates for CRT.

### Study limitations

The CC alterations evaluated in this study pertain to individuals without structural heart disease or rhythm disturbances, such as atrial arrhythmia or advanced atrioventricular conduction block. These findings need to be validated in other clinical conditions, such as previous myocardial infarction, LV dysfunction, severe valvular diseases, or other situations that were not part of this study’s inclusion criteria.

## Conclusions

The analysis of the four intraventricular conduction disorder patterns demonstrated significant associations with CC and ventricular synchrony changes, varying in degree and location according to the block type. The main change observed in the CC was the increase in LV pre-ejection period, associated with both LBBB and RBBB+LAFB. Only the LBBB pattern showed a significant association with left intraventricular dyssynchrony.
